# Plymouth Borough Hospital

**Published:** 1898-09-24

**Authors:** 


### THE HOSPITAL SUNDAY FUND.

### PLYMOUTH BOROUGH HOSPITAL.

At the last meeting of the Plymouth Board of Guardians some very serious charges were brought against the management of the Plymouth Borough Hospital. A committee had been appointed to inquire into the treatment of the children who had been sent to the hospital from the workhouse, and for whose treatment and maintenance the guardians paid £1 1s. a week to the sanitary authority. They reported that they had received complaints as to the condition of one of the children to the effect that it was found by the mother to be "dirty, wet, neglected, and very cold. She found its milk bottle very dirty, as also was its chemise, and it had a large bruise on its back the size of half-a-crown." Both this child and others in the wards were without night dresses, the excuse being that they "had committed themselves." The committee have also made inquiries "to ascertain whether it is a fact that out of twelve children who recently returned to the workhouse from the Infectious Diseases Hospital, eight were found on admission to the house to be suffering from vermin in their heads, and some from sores, and from evidence which the committee have obtained they find that it is a fact."

Complant was also made in the report that, although the children sent to the hospital are paid for, "they are spoken of by the officials to the friends of the children as 'pauper children.'"

Other complaints were made as to the children being detained an unnecessarily long time in the hospital, but when we hear the case of one child who had been detained "11 weeks," so that the Board had had "to pay 11 guineas," quoted as an extreme example, we would remind the Guardians that, as things go at many fever hospitals, that is by no means an out of the way length of stay. In regard to the other matters, however, there can be no doubt that a most careful investigation should be undertaken by the Sanitary Committee of the Plymouth Corporation. To a large extent fever hospitals are dark places, so far as outside observation is concerned. Naturally and properly, the visits of friends are restricted as far as possible, and those in actual charge of the patients are but little brought under the eye of the general public. Greatly to the credit of the officials, it should be said that in the majority of cases the patients are well treated and carefully looked after. But constant vigilance should be exercised by all sanitary committees in regard to their fever hospitals, for the patients are mostly little children, and when any laxity of administration creeps in things may go very wrong indeed within the walls before anything is heard of them outside.

